# Gastrointestinal bleeding during the transcatheter aortic valve replacement perioperative period: A Review

**DOI:** 10.1097/MD.0000000000031953

**Published:** 2022-12-02

**Authors:** Chuan Lu, Yue Zhang

**Affiliations:** a From the Second Hospital of Dalian Medicial University, Shahekou District, Dalian City, China.

**Keywords:** gastrointestinal bleeding, perioperative period, TAVR

## Abstract

With the aging of the population, the incidence of senile degenerative valvular heart disease is expected to increase. Transcatheter aortic valve replacement (TAVR) has been used for patients at lower surgical risk with symptomatic severe aortic valve stenosis. Because of the improvements in TAVR technology and increasing experience of the operators, TAVR is regarded as a safe and feasible procedure. Bleeding events during the TAVR perioperative period, especially gastrointestinal (GI) bleeding, have been proven to be related to the long-term prognosis and mortality. Elderly patients with valvular heart disease are susceptible to GI bleeding because of their use of antithrombotic drugs, physical damage of coagulation factors, and GI angiodysplasia. Frequent GI bleeding and low levels of preoperative hemoglobin increase the risk of TAVR, especially for elderly patients. Because of these risks, which are easily overlooked, we should focus more attention on the perioperative management of TAVR. Reasonable screening tools, including blood examinations, risk evaluation scales, and endoscopy, are beneficial to the prevention of complications that can occur during the perioperative period. Additionally, medical therapy can safely help patients at high-risk for bleeding patients throughout the perioperative period. This study aimed to characterize the pathology of TAVR patients and discuss treatment strategies for GI bleeding during the perioperative period.

## 1. Introduction

Aortic valve stenosis (AVS) is a valvular heart disease with poor treatment effects and a poor prognosis. The incidence of AVS has increased recently because of the aging of the population. Conservative therapy has a poor treatment effect on severe AVS, and aortic valve replacement is regarded as a measure that can eliminate AVS. Different from surgical aortic valve replacement, transcatheter aortic valve replacement (TAVR) is initially used for patients with symptomatic severe aortic valve stenosis, especially those at intermediate or high surgical risk.^[1]^ Currently, TAVR has been expanded to patients at low surgical risk by some researchers based on the evidence of several large-sample, randomized, clinical trials.^[2–5]^ Bleeding events are the most common complications of TAVR, and they can occur during the early and late periods.^[6,7]^ Late bleeding (>30 days), which frequently involves gastrointestinal (GI) bleeding, is recognized as one of the indicators of the long-term prognosis and mortality of TAVR patients.^[8]^ Currently, the most common type of perioperative bleeding is access site-related bleeding.^[7]^ With developments of medical devices and operating levels, the incidence of access site-related bleeding has been decreased, and we should pay more attention to other types of perioperative bleeding. Unfortunately, even though GI bleeding during the perioperative period is one of the main life-threatening complications, it is often ignored compared with other complications. This study aimed to characterize the pathology of TAVR and methods of detecting TAVR and to discuss treatment strategies for GI bleeding during the perioperative period.

## 2. Pathophysiology

### 2.1. High shear rate and coagulopathy

Patients with severe AVS are more likely to experience bleeding events, such as GI bleeding or other organic types of bleeding. As reported by previous studies, there is a causal association between AVS and coagulopathy that is attributed to the loss of von Willebrand factor (VWF).^[9]^ VWF is a multimeric globular plasma glycoprotein produced by endothelial cells and megakaryocytes that is stored in Weibel-Palade bodies and alpha granules of platelets.^[9–11]^ The function of VWF is to perform hemostasis and mediate platelet adhesion to injured vessels.^[12]^ High shear rate caused by calcified and stenotic aortic valves changes the structure of VWF into an elongated form that is sensitive to the specific action of ADAMTS 13, which is a von Willebrand protease.^[13]^ Consequently, this leads to the loss of VWF high-molecular-weight multimers and platelet-mediated hemorrhage.^[14,15]^ This type of acquired von Willebrand disease (aVWD) ascribed to high shear rate is known to occur in patients with AVS, hypertrophic cardiomyopathy, and mitral and aortic insufficiency.^[16]^

### 2.2. Angiodysplasia and Heyde syndrome

Angiodysplasia is a common cause of GI bleeding, especially for patients older than 60 years.^[17]^ There are correlations among angiodysplasia, cardiac valve calcification, and age.^[18]^ According to their epidemiological characteristics, angiodysplasia and valve calcification seem to be signs of senility. This characteristic syndrome can be found in elderly patients. The first case of GI bleeding with AVS was described by Dr Edward Heyde in 1958; since then, it has become known worldwide. Heyde syndrome (HS) is characterized by AVS, GI bleeding caused by GI angiodysplasia, and aVWD. It is difficult to determine the accurate incidence of HS because it is not well-understood and is difficult to diagnosis. Approximately 3% of AVS patients experience recurrent GI bleeding, supported by some observations.^[19]^ When this occurs, it is possible to approximately calculate the incidence of HS. Because of the lack of routine preprocedural detection, including endoscopy and VWF activity, it is possible that the true incidence of HS is underestimated. Although a large proportion of AVS patients are not definitely diagnosed with HS, they are known to be susceptible to GI bleeding.

Currently, it is considered that there is a link between AVS and GI bleeding. Degenerative valvular heart disease and GI angiodysplasia usually appear in older individuals; therefore, they can be categorized as age-related degeneration.^[20]^ Furthermore, these factors can be considered signs of senescence. It has been hypothesized that GI mucosal ischemia secondary to low cardiac output that occurs with severe AVS is the leading cause of recurrent bleeding.^[21,22]^ There is growing evidence that low-level VWF caused by high shear rate increases the vulnerability to bleeding.^[20]^ The pathophysiology of HS is multifactorial, and the relationship between AVS and angiodysplasia remains controversial. Because AVS patients are susceptible to GI bleeding, it is necessary to focus more attention on the prophylaxis and treatment of perioperative GI bleeding.

### 2.3. Assessment

#### 2.3.1. *Endoscopy*.

GI angiodysplasia is the pathological communication between mucosal capillaries and submucosal veins.^[23]^ Pathological lesions usually appear in patients older than 60 years; therefore, they are recognized as an age-related degenerative process. Angiodysplasia can be found throughout the entire GI tract; however, the right colon (especially the ascending colon and cecum) is the most common location. However, some recent studies reported conflicting results. Bollinger et al performed a retrospective analysis and found that most cases of GI angiodysplasia were in the proximal small bowel instead of the right colon.^[24,25]^ More evidence is required to validate this point of view in the future.

Endoscopy is currently the gold standard modality for diagnosing GI angiodysplasia, which is characterized by focal or diffused, cherry red lesions with venous dilatation radiating from a feeding vessel.^[23,26]^ Commonly, GI angiodysplasia involves multiple sites and more than 1 lesion across the entire GI tract.^[27]^ It is difficult to find all the culprit lesions in patients with recurrent GI bleeding.Fortunately, with the development of digestive endoscopy (especially capsule endoscopy and balloon-assisted enteroscopy) and imaging technology, the detection rate of GI angiodysplasia has greatly improved. Therefore, they can be broadly applied for endoscopic treatment.

Undoubtedly, appropriate endoscopic testing of specific individuals, including those with HS and unexplained anemia, is a valuable strategy. Because of the development of endoscopic techniques, it is not difficult to make a correct diagnosis of GI bleeding in most cases. However, in the case of angiodysplasia-related GI bleeding, even experienced operators can miss some concealed lesions. Therefore, repeated examinations are required, particularly for patients with unexplained anemia and a history of recurrent GI bleeding.

#### 2.3.2. *Laboratory assays*.

It is of great importance to evaluate the bleeding risk during the perioperative period. Perioperative bleeding in AVS patients can lead to a poor prognosis and cause fatal outcomes. Considering the poor tolerance to anemia, it is essential to evaluate the risk in advance to avoid perioperative bleeding. In addition to conventional detection methods, such as the evaluation of hemoglobin levels, platelet counts, and coagulation function, more specific methods are necessary for AVS patients.

The activity of VWF multimers is a sensitive indicator of the tendency for hemorrhage in AVS patients. Soon after aortic valve replacement, high-molecular-weight VWF multimer levels recover and GI bleeding stops in many cases.^[28,29]^ Although measuring the activity of VWF multimers is the gold standard for diagnosing aVWD, the complexity and time consumption of this method hinder its wide application in routine laboratories.^[30]^

The platelet function analyzer is a sensitive assay of high-molecular-weight multimers that is advantageous because it is simple to use, requires little blood, and can be performed at the point of care.^[31,32]^ Closure time adenosine diphosphate (CT-ADP), which is an important index assessed using the platelet function analyzer, can sensitively reflect platelet function.^[33]^ Although the CT-ADP has poor diagnostic specificity for aVWD and is merely recognized as a screening tool, it is widely applied to assess the bleeding risk of AVS patients. Several studies have indicated relationships between the prolongation of CT-ADP (>180 seconds) and 1-year mortality, major life-threatening bleeding, and paravalvular aortic regurgitation after TAVR.^[33–35]^ Furthermore, it appears to be a highly cost-effective method of evaluating the coagulation function of TAVR patients and is worthy of extensive use.

#### 2.3.3. *Bleeding assessment tools*.

Assessing the risk of perioperative bleeding is important; therefore, many risk score questionnaires, such as the Society of Thoracic Surgeons score and Euro SCORE II score, have been widely used to predict the mortality of patients undergoing cardiac surgery.^[36–38]^ Additionally, there are some questionnaires for evaluating the degree of GI bleeding, such as the Rockall score and Glasgow Blatchford score. Additionally, it is necessary to assess the coagulation function. The ISTH Bleeding Assessment Tool (ISTH-BAT) is an effective and convenient screening tool that is frequently used for this purpose.

Although the ISTH-BAT is mainly utilized for diagnosing blood diseases, it is also suitable for assessing coagulopathy ascribed to AVS and other valvular heart diseases. It is not difficult to aggregate the specific score when using the ISTH-BAT to evaluate different bleeding symptoms. The ISTH-BAT scores of 4 for males and 6 for females are regarded as signs of potential bleeding.^[39]^ Because the scores are related to the severity of bleeding and VWF levels,^[40]^ these questionnaires seem especially suitable for perioperative AVS patients and will help physicians perform more comprehensive assessments and develop individualized antithrombotic therapy for high-risk patients.

#### 2.3.4. *Others*.

Helical computed tomography angiography and magnetic resonance angiography are conventional diagnostic methods.^[41,42]^ When the diagnosis is unclear, combining these with endoscopy can improve the detection sensitivity. Standard angiography is widely used for cardiovascular disease and occasionally used for acute bleeding. However, its application for GI bleeding is limited.

### 2.4. Examination strategies

Although AVS patients are at higher risk for GI bleeding than others, endoscopy is not required for all patients. It is necessary to emphasize noninvasive or less invasive screening for high-risk groups during the perioperative period, such as complete blood count testing, routine coagulation testing, use of the platelet function analyzer, fecal occult blood testing, and use of bleeding questionnaires, which are effective and convenient methods.

Additionally, baseline comorbidities, such as female sex, old age, chronic kidney disease, liver disease, atrial fibrillation, and history of GI bleeding, are significant predictors of perioperative adverse events.^[43–45]^ When encountering patients with these characteristics, it is essential to perform more detailed evaluations to reduce perioperative adverse events. Definitive diagnostic and therapeutic strategies should be developed by physicians and surgeons working together.

### 2.5. Treatment strategies

There are many different causes of GI bleeding that can be treated by different therapies. Malignant tumors, hepatic cirrhosis, and bleeding caused by trauma are not discussed in this paper. Peptic ulcers are the most common cause of upper GI bleeding, and nonsteroidal anti-inflammatory drugs are generally used for these patients. They usually have a good prognosis with the application of conventional drug therapy and adjustment for nonsteroidal anti-inflammatory drugs. Diverticular disease is a common reason for lower GI bleeding, which has an incidence exceeding 20% among hospitalized patients.^[46]^ Current global clinical guidelines recommend mechanical therapy, such as thermal coagulation, endoscopic clipping, and endoscopic ligation.^[47]^ Through mechanical therapy or drug therapy, patients with GI bleeding can commonly achieve a stable condition. Furthermore, patients with preferable conditions can safely undergo TAVR.

Angiodysplasia is another cause of GI bleeding that is common in elderly individuals. As 1 characteristic of HS in TAVR patients, angiodysplasia requires more attention. The incidence of degenerative valvular heart disease has been increasing in elderly individuals in recent years. These patients usually have GI angiodysplasia or unexplained anemia as well, but only a few of them are definitively diagnosed with HS. Therefore, when treating patients with these features, the possibility of HS must be considered and individualized therapy must be provided. Conservative management and aortic valve replacement for patients with suspected or proven HS have been debated. Currently, there is a lack of definite evidence regarding which is preferrable, and only some case reports and small-scale trials have been published (Table 1).

**Table 1:** Studies showing effects TAVR/SAVR on HS.

Author	Yr	Types of study	Types of AVR	Total/HS	Follow-up	Results
Thompson et al^[18]^	2012	Retrospective	SAVR	57	15yrs	45 patients (79%) had no recurrence of GI bleeding after SAVR,12 patients (21%) had recurrence of GI bleeding.
Godino et al^[48]^	2013	Retrospective	TAVR	400/7	22 ± 15mo	6 patients (85%) had no recurrence of GI bleeding,1 patient(TAVR failed) had transfusion–dependent anemia.
Caspar et al^[49]^	2015	Prospective	TAVR	40/1	1 yr	HS patients’ hemostatic parameters improved after TAVR, had no recurrence of GI bleeding during 1 year follow-up. vWF of all patients improved 1 week after TAVR.
Sedaghat et al^[50]^	2017	Prospective	TAVR	74/2	1 yr	30 patients (48.6%) had a loss of HMW VWF multimers before TAVR, and they were restored after TAVR.
Waldschmidt et al^[51]^	2021	Retrospective	TAVR	2548/47	11.2 mo	The recurrence of GI bleeding in HS patients was higher (39.8%) compared to non-HS patients (21.2%); The rate of PVL after TAVR was higher in HS patients.

GI = gastrointestinal, HS = Heyde syndrome, PVL = paravalvular leakage, SAVR = surgical aortic valve replacement, TAVR = transcatheter aortic valve replacement, VWF = von Willebrand factor.

A retrospective observational study of a cohort of 400 TAVR patients was conducted by Godino et al, who noticed that the incidence of HS is not rare (1.7%) and that GI bleeding is resolved after successful TAVR.^[48]^ Thompson et al observed similar results when evaluating the effectiveness of surgical aortic valve replacement for reducing GI bleeding in HS patients. Forty-five patients (79%) showed no recurrence of GI bleeding after aortic valve replacement during 15 years of follow-up.^[18]^ Garcia et al reported a case of GI bleeding treated by conventional aortic valve replacement with a mechanical 23-mm optimized double leaflet prosthesis.^[52]^ Alshuwaykh et al reported that GI bleeding in a female patient improved with AS and small bowl angiodysplasia after endoscopic therapy and TAVR; however, melena recurred with a hemoglobin level of 7.6 g/dL after 2 weeks, and recovery was achieved with conservative treatment.^[53]^ During 1 to 6 months of follow-up, no episodes of GI bleeding were observed.^[53]^ Additionally, the disappearance of GI angiodysplasia after TAVR has been reported.^[54]^ Therefore, some studies have reported recurrent GI bleeding as an indication for TAVR in HS patients.

However, the latest evidence provided by a single-center, retrospective study showed the opposite result. A cohort of 2548 consecutive patients who underwent TAVR were divided into 2 groups, the Heyde group and the non-Heyde group. The diagnostic criteria for HS comprised a clinical triad of severe AS, a history of recurrent GI bleeding, and an endoscopic diagnosis of angiodysplasia. During 1 year of follow-up after TAVR, the recurrence of GI bleeding was higher for Heyde patients (39.8%) than for non-Heyde patients (21.2%). Furthermore, the researchers noticed that the rate of paravalvular leakage (PVL) after TAVR was higher for HS patients than for other groups.^[51]^

It is well-known that PVL has been proven to be the strongest predictor of bleeding events between 30 days and 1 year after TAVR.^[35]^ Therefore, only some patients with GI bleeding ascribed to angiodysplasia can be successfully treated with TAVR. Because of PVL and late bleeding complications, these patients should be monitored more carefully during the perioperative period. Therefore, it is worth considering whether TAVR is suitable for HS patients. However, the aforementioned study was limited because aVWD was not included in the diagnostic criteria for HS, which might have affected the final results. More evidence supported by a multitude of prospective studies is necessary.

### 2.6. Medical treatment

Medical treatment is also necessary during the perioperative period. Intensive therapy will benefit patients with GI bleeding and those at high risk for bleeding. Compared with other treatment options, medical therapy has better efficacy, results in fewer injuries, is safer, and is associated with fewer side effects, thus leading to good clinical outcomes. Different types of remedies are applicable to patients with different conditions of the GI tract (Table 2).

**Table 2:** Medicine treatment.

Product	Mechanism of action	Adverse effect
PPIs	Inhibit gastric acid secretion	Diarrhea, headache pay attention to the combination with other CYP450 related agents
Desmopressin	Releases endogenous VWF, FVIII	Fatigue, headache, nausea, hypertension
Tranexamic acid	Antifibrinolytic agents	Epilepsy, gastrointestinal symptoms
Octreotide	Reduce visceral blood flow, suppress digestive enzymes, inhibite angiogenesis	Gastrointestinal symptoms, hypertension
Thalidomide	Inhibite angiogenesis	Teratogenicity, peripheral neuropathy, sedation, rash, constipation
Estrogen/Progesterone	Integrate blood vessel endothelium	Thromboembolism, hypertension, tumor
Alphanate	VWF/FVII concentrates	Thromboembolism, expensive
Vonvendi	recombinant vWF	Thromboembolism, expensive
Platelet	Platelet activation and aggregation	Transmission of infection, fever, hemolysis, anaphylaxis
Fresh frozen plasma	Contains VWF	Transmission of infection, fever, hemolysis, anaphylaxis
Cryoprecipitate	Contains VWF	Transmission of infection, fever, hemolysis, anaphylaxis

PPIs = proton pump inhibitors, VWF = von Willebrand factor.

#### 2.6.1. *Proton pump inhibitors*.

Proton pump inhibitors (PPIs) are among the most commonly used inhibitory gastric acid drug. PPIs therapy has been proven to reduce the incidence of hospitalization for upper GI bleeding among patients receiving oral anticoagulation.^[55]^ Scally et al performed a meta-analysis of randomized trials and found that PPIs could reduce the risk of peptic ulcers and their complications.^[56]^ PPIs are recommended for preventing and treating GI bleeding by numerous clinical guidelines. Therefore, they seem applicable to patients undergoing TAVR, who frequently rely on antithrombotic therapy. PPIs, with their high efficacy and minimal side effects, represent an essential part of modern gastroenterology. If there are no contraindications, then patients undergoing TAVR should use PPIs before and after surgery.

#### 2.6.2. *Treatment for VWF*.

The loss of high-molecular-weight multimers because of high shear rate in patients with severe AVS is common. Although the VWF level will recover soon after TAVR, low VWF levels can increase the risk of bleeding during the perioperative period. In general, there are 2 ways to increase the VWF level: improving the internal release of VWF and providing external VWF.

#### 2.6.3. *Desmopressin*.

Desmopressin (1-deamino-8-d-arginine vasopressin) is a synthetic analogue of the antidiuretic hormone vasopressin. It can facilitate the release of VWF and coagulation factor VIII from vascular endothelial cells. Currently, it is mainly recommended as an effective method for congenital bleeding disorders. Additionally, desmopressin is used for perioperative bleeding and trauma according to some guidelines.^[57]^ Compared with hematological disease, suitable patients are those with severe valvular heart diseases who are of advanced age and use antiplatelet agents. With the improvement of the coagulation function after using desmopressin, the balance may shift from bleeding to thrombus. Therefore, it is necessary to consider not only the prevention of bleeding but also the incidence of thrombotic events. Desborough reported evidence of reversal treatment with antiplatelet agents after finding that desmopressin is an effective treatment for preventing perioperative bleeding, and that it does not increase the risk of thrombotic events or mortality, as evidenced by a meta-analysis of randomized controlled trials; however, large-sample trials are required to confirm this, especially for TAVR patients.^[58]^ In conclusion, it is necessary to weigh the benefits and risks for patients undergoing TAVR before administering desmopressin.

#### 2.6.4. *External therapeutics*.

The direct method of improving the coagulation function is the transfusion of blood products, such as cryoprecipitate or fresh-frozen plasma. It is clear that these types of blood products are rich in diverse coagulation factors; therefore, they are frequently used during perioperative period. Cardiac function should be considered during the application of transfusions because AVS patients usually have New York Heart Association class III or IV heart failure. Additionally, some researchers have suggested using VWF/ coagulation factor VIII concentrates and recombinant VWF (Alphanate and Vonvendi) to prevent bleeding, which have been used for aVWD. However, there is no evidence to support their use. Caution is required when considering these therapies for TAVR patients because of their high thromboembolic risks.^[59]^

#### 2.6.5. *Antifibrinolytic agents*.

Antifibrinolytics can inhibit fibrinolysis and help prevent bleeding with trauma, surgery, and non-surgical diseases. Tranexamic acid (TXA) is an antifibrinolytic agent that competitively inhibits the activation of plasminogen to plasmin. It has been used for many disorders, such as von Willebrand disease and GI bleeding, and to guard against bleeding after surgery; furthermore, numerous studies have found evidence supporting its use. Lee et al reported that TXA is an effective treatment for upper GI bleeding and emphasized its early administration.^[60]^ Similarly, antifibrinolytic agents have been known to induce blood loss during cardiac surgery. Considering the risk of cardiovascular and cerebral vascular events, the choice of antifibrinolytics for cardiac surgery has shifted from aprotinin to TXA.^[61]^ Evidence of the success of TXA for hemorrhage is available; however, there have not been any large-sample trials involving TAVR patients.

#### 2.6.6. *Treatment for angiodysplasia*.

Different treatment is used for GI bleeding caused by angiodysplasia. Angiodysplasia has the characteristics of latency and frequent recurrence in elderly patients, and patients with severe AVS have poor tolerance to anemia. Recurrent GI bleeding is recognized as one of the indications for TAVR for patients with HS or suspected HS. Endoscopic or conservative treatment is less effective according to long-term follow-up, which showed a high readmission rate.^[62]^ Although not a curative method, medical treatment has an important role in bridging to cardiac surgery.

#### 2.6.7. *Octreotide*.

Octreotide has been used for the treatment of acromegaly and gastro-entero and pancreatic neuroendocrine tumors for many years. It is currently the first-line agent for the treatment and secondary prevention of GI bleeding. As a somatostatin analog, octreotide can reduce the visceral blood flow, decrease portal venous pressure, suppress digestive enzymes, and inhibit angiogenesis in the GI tract through downregulation of vascular endothelial growth factor.^[63]^ Based on this mechanism, octreotide is believed to reduce GI bleeding rates caused by angiodysplasia.

The treatment of GI bleeding for patients using left ventricular assist devices (LVAD) has been a popular topic in recent years. During a multicenter and retrospective study, Shah et al found that secondary prophylaxis with octreotide can reduce the GI bleeding rates of patients using LVAD compared with a control group who did not receive octreotide (24% vs 43%).^[64]^ Wilson et al reported a similar decrease in GI bleeding events with octreotide treatment. Nineteen patients (59.4%) did not have subsequent GI bleeding after using octreotide. The frequency of bleeding events for the other 13 patients was 2.6 per patient per year, which was significantly lower than the historical level.^[65]^

Similar to the pathophysiology of patients using LVAD, the risk factors for an increased likelihood of GI bleeding for patients with severe AVS include the loss of VWF secondary to high shear rate, application of antiplatelet and anticoagulant agents, angiodysplasia in the GI tract, and intestinal hypoperfusion. Therefore, octreotide is also considered suitable for AVS patients. Prophylaxis treatment seems to be a valuable method that can reduce the risk of GI bleeding and provide patients with a better status before surgery. However, the side effects of octreotide, such as hypertension, bradycardia, and hyperglycemia, should be considered.

#### 2.6.8. *Thalidomide*.

Thalidomide is an antiangiogenic, antitumor, anti-inflammatory, and immunomodulating drug.^[66]^ Because of the effects of its antiangiogenic activity, it has been used to treat recurrent GI bleeding caused by vascular malformations for nearly 10 years. The first formal prospective study of thalidomide treatment for GI bleeding caused by angiodysplasia was reported in 2011.^[67]^ Ge et al found that thalidomide appears to be an effective and relatively safe treatment for patients with GI bleeding caused by vascular malformations. During follow-up (mean, 39 months), the recurrence rate of bleeding episodes was significantly decreased for patients with thalidomide (total of 28 patients), and no severe adverse effects were observed. These are encouraging results. Chen et al reported similar results in 2016 after performing a retrospective study to observe the long-term response and adverse effects of thalidomide experienced by patients with GI bleeding. The mean follow-up period was 42.6 months, and the overall response rate was 77.5% (62 of 80 patients).^[68]^ Thalidomide appears to be an effective treatment for GI bleeding caused by angiodysplasia. Additionally, some case reports have indicated the good response of recurrent GI bleeding with continuous-flow LVAD.^[69,70]^

Although thalidomide is effective, its adverse effects cannot be ignored. Thalidomide is associated with peripheral neuropathy, sedation, rash, and constipation. However, its most notorious side effect is teratogenicity, similar to chemotherapeutic agents. Fortunately, patients with AVS are usually beyond child-bearing age and have no fertility requirements. Therefore, teratogenicity is not an important issue for these patients.

### 2.7. Estrogen-based hormone therapy

Hormone therapy has been recognized as a potentially useful treatment for patients with bleeding caused by multiple angiodysplastic lesions. Estrogen or estrogen combined with progesterone can theoretically prevent bleeding by improving the integrity of the blood vessel endothelium. However, some existing studies do not support this theory. The most remarkable study was a multicentered, randomized, controlled trial reported by Junquera et al.^[71]^ In conclusion, hormone therapy does not effectively prevent recurrent bleeding caused by angiodysplasia. Additionally, thromboembolism with estrogen-based therapies should be considered when applied to patients with cardiovascular diseases. Therefore, this therapy is not necessarily the first choice for patients undergoing TAVR.

### 2.8. Transfusion strategy

It is essential to maintain an appropriate level of hemochrome for patients undergoing TAVR during the perioperative period. Blood transfusion appears to be a beneficial method of increasing the safety of surgery. However, some studies have reported the deleterious effects of red blood cell transfusion after cardiovascular surgery. Nuis et al found that blood transfusion was related to the occurrence of acute kidney injury after TAVR.^[72]^ More than 1 center has reported increased rates of major strokes and 1-year mortality after TAVR patients received blood transfusions.^[73,74]^ Therefore, it is imperative to judge the benefits and risks carefully before formulating a blood transfusion strategy.

With improvements in interventional techniques and medical devices, the rate of site-related bleeding has greatly decreased. More attention must be focused on the risk of GI bleeding during the perioperative period. However, the benefits and risks of blood transfusion therapy remain controversial. Pretreatment of anemia before TAVR, such as iron substitution and erythropoietin, might be a feasible approach.

## 3. Conclusions

It is necessary to focus close attention on the susceptibility to and risk of GI bleeding for AVS patients, especially during the perioperative period. Furthermore, it is essential to emphasize detection methods before TAVR. Adequate preoperative assessment and rational pretreatment are indispensable methods of ensuring the safety of TAVR.

## Author contributions

**Writing – original draft:** Chuan Lu.

**Writing – review & editing:** Yue Zhang.
